# Exploring the efficacy of multi-flavored feature extraction with radiomics and deep features for prostate cancer grading on mpMRI

**DOI:** 10.1186/s12880-023-01140-0

**Published:** 2023-11-22

**Authors:** Hasan Khanfari, Saeed Mehranfar, Mohsen Cheki, Mahmoud Mohammadi Sadr, Samir Moniri, Sahel Heydarheydari, Seyed Masoud Rezaeijo

**Affiliations:** 1 Department of Mechanical Engineering, Petroleum University of Technology, Ahvaz, Iran; 2 Department of Electrical Engineering, Amirkabir University of Technology, Tehran, Iran; 3 Department of Medical Imaging and Radiation Sciences, Faculty of Paramedicine, Ahvaz Jundishapur University of Medical Sciences, Ahvaz, Iran; 4 Department of Medical Physics, School of Medicine, Isfahan University of Medical Sciences, Isfahan, Iran; 5 Department of Medical Physics, Faculty of Medicine, Ahvaz Jundishapur University of Medical Sciences, Ahvaz, Iran; 6 Cancer Research Center, Ahvaz Jundishapur University of Medical Sciences, Ahvaz, Iran

**Keywords:** Radiomics features, Deep features, Grading, Prostate cancer, mpMRI

## Abstract

**Background:**

The purpose of this study is to investigate the use of radiomics and deep features obtained from multiparametric magnetic resonance imaging (mpMRI) for grading prostate cancer. We propose a novel approach called multi-flavored feature extraction or tensor, which combines four mpMRI images using eight different fusion techniques to create 52 images or datasets for each patient. We evaluate the effectiveness of this approach in grading prostate cancer and compare it to traditional methods.

**Methods:**

We used the PROSTATEx-2 dataset consisting of 111 patients’ images from T2W-transverse, T2W-sagittal, DWI, and ADC images. We used eight fusion techniques to merge T2W, DWI, and ADC images, namely Laplacian Pyramid, Ratio of the low-pass pyramid, Discrete Wavelet Transform, Dual-Tree Complex Wavelet Transform, Curvelet Transform, Wavelet Fusion, Weighted Fusion, and Principal Component Analysis. Prostate cancer images were manually segmented, and radiomics features were extracted using the Pyradiomics library in Python. We also used an Autoencoder for deep feature extraction. We used five different feature sets to train the classifiers: all radiomics features, all deep features, radiomics features linked with PCA, deep features linked with PCA, and a combination of radiomics and deep features. We processed the data, including balancing, standardization, PCA, correlation, and Least Absolute Shrinkage and Selection Operator (LASSO) regression. Finally, we used nine classifiers to classify different Gleason grades.

**Results:**

Our results show that the SVM classifier with deep features linked with PCA achieved the most promising results, with an AUC of 0.94 and a balanced accuracy of 0.79. Logistic regression performed best when using only the deep features, with an AUC of 0.93 and balanced accuracy of 0.76. Gaussian Naive Bayes had lower performance compared to other classifiers, while KNN achieved high performance using deep features linked with PCA. Random Forest performed well with the combination of deep features and radiomics features, achieving an AUC of 0.94 and balanced accuracy of 0.76. The Voting classifiers showed higher performance when using only the deep features, with Voting 2 achieving the highest performance, with an AUC of 0.95 and balanced accuracy of 0.78.

**Conclusion:**

Our study concludes that the proposed multi-flavored feature extraction or tensor approach using radiomics and deep features can be an effective method for grading prostate cancer. Our findings suggest that deep features may be more effective than radiomics features alone in accurately classifying prostate cancer.

## Introduction

Prostate cancer is a prevalent form of cancer among men, second only to lung cancer in terms of incidence. It is estimated that there will be approximately 174,650 new cases and 31,620 deaths related to prostate cancer in the United States in 2019 [1]. This represents a significant proportion of all new cancer diagnoses among men, accounting for approximately one in five cases. Early detection and treatment planning are crucial in reducing the mortality rate associated with prostate cancer [1–3]. Therefore, healthcare professionals must be aware of the risk factors and implement screening and diagnostic protocols for prostate cancer. Additionally, treatment planning should be individualized based on the patient’s risk profile, taking into consideration the patient’s age, comorbidities, and preferences [4–6].

Traditionally, prostate cancer is diagnosed through a combination of digital rectal examination (DRE) and prostate-specific antigen (PSA) blood test, followed by transrectal ultrasound (TRUS) guided sampling. The diagnosis is based on the microscopic evaluation of prostate tissue obtained through needle sampling. Currently, the gold standard for diagnosing prostate cancer is prostate sampling under the guidance of TRUS [7]. A pathologist reviews these samples and assigns a primary Gleason score for the predominant histological pattern and a secondary Gleason score for the worst pattern [8]. The Gleason Score (GS) serves as a standard indicator of the aggressiveness of prostate cancer. It is determined by adding the two most prevalent Gleason grades found in the cancerous tissue pattern. Pathologists assign a Gleason grade, which ranges from 3 to 5, based on the arrangement of cancer cells within the prostate. Prostate cancer is divided into five distinct grade groups, depending on the Gleason Score. A Gleason Score less than 6 falls into Grade Group 1 (GG 1), while a Gleason Score of 7 or higher is placed in Grade Group 2 (GG 2, GS 3 + 4 = 7), Grade Group 3 (GG 3, GS 4 + 3 = 7), Grade Group 4 (GG 4, GS 4 + 4 = 8, GS 3 + 5 = 8, GS 5 + 3 = 8), and Grade Group 5 (GG 5, GS = 9, GS = 10) based on the level of cancer aggressiveness [9]. Doctors make predictions about the likelihood of recovery based on the grade group. Lesions classified as GG 1 are generally considered insignificant from a clinical standpoint and do not necessitate treatment. However, active surveillance is recommended for such lesions. In contrast, lesions categorized as GG 2, GG 3, GG 4, and GG 5 are deemed clinically significant and typically require treatment.

Computer-aided diagnosis (CAD) techniques have been proposed as a means of assisting radiologists in determining the grade of prostate cancer from magnetic resonance imaging (MRI) scans [10–12]. Recently, several studies have focused on the classification of clinically significant and clinically insignificant prostate cancer. Methods utilizing texture feature analysis and Convolutional Neural Networks (CNNs) have shown promising results [13–16]. The use of computer-aided quantitative analysis for prostate multiparametric MRI (mpMRI) has the potential to improve the detection of prostate cancer and aid in standardizing mpMRI interpretation. This, in turn, could lead to a more efficient diagnostic process and reduce the number of over and under-diagnoses in prostate cancer management. However, various methods have been proposed for identifying significant prostate cancer using deep learning networks or radiomics approaches. Differences in patient population, dataset size, imaging protocols, and other factors make it difficult to compare the performance of these methods [17–20].

The aim of this research is to investigate the use of radiomics features and deep features obtained from multiparametric magnetic resonance imaging (mpMRI) to grade prostate cancer. We propose a novel approach in which four mpMRI images (T2 weighted image (T2W) – transverse, T2W-sagittal, Diffusion-weighted imaging (DWI), and apparent diffusion coefficient (ADC)) are combined using eight different fusion techniques, creating 52 images or datasets for each patient. This approach, referred to as multi-flavored feature extraction or tensor, has not been previously explored. Our work addresses several limitations of previous methods in grading prostate cancer using deep learning networks or radiomics approaches. Specifically, most previous studies focus on the classification of clinically significant and clinically insignificant prostate cancer using a single imaging modality or the fusion of two modalities, which may not capture the complete information about the tumor. In contrast, our approach uses multiple imaging modalities and fusion techniques to extract complementary information from different aspects of the tumor, which may improve the accuracy of prostate cancer grading. Furthermore, we compare the performance of our approach to traditional methods, providing insights into the potential benefits of our approach for prostate cancer diagnosis and management.

The main contributions are shown as follows:

- Proposing a novel approach, called multi-flavored feature extraction or tensor, that combines four mpMRI images using eight different fusion techniques to extract complementary information from different aspects of the tumor. This novel approach has not been previously explored.
- Investigating the use of radiomics features and deep features obtained from mpMRI to grade prostate cancer, which has the potential to improve the accuracy of prostate cancer diagnosis and management.
- Addressing several limitations of previous methods in grading prostate cancer using deep learning networks or radiomics approaches, such as the focus on the classification of clinically significant and clinically insignificant prostate cancer using a single imaging modality or fusion of two modalities. Our approach uses multiple imaging modalities and fusion techniques to extract complementary information from different aspects of the tumor, which may improve the accuracy of prostate cancer grading.
- Comparing the performance of our approach to traditional methods, which provides insights into the potential benefits of our approach for prostate cancer grading.

### Related work

Previous research in the field of prostate cancer has predominantly concentrated on the categorization and grading of prostate tumors. In this section, we present a concise overview of the existing literature pertaining to these key aspects. Liu et al. [21]. delved into the exploration of whether the amalgamation of radiomics and automated machine learning-based classification, particularly for the original images obtained from multiphase dynamic contrast-enhanced (DCE)-MRI scans, could accurately forecast the aggressiveness of prostate cancer before resorting to a biopsy procedure. Their findings revealed that a fusion of radiomics and machine learning-driven analysis, focusing on the earliest and most robust phases of the original DCE-MRI images, could non-invasively and precisely predict prostate cancer aggressiveness. In a similar vein, Castillo et al. [18]. undertook a comparative analysis, evaluating the performance of a deep-learning model against that of a radiomics model in diagnosing significant prostate cancer within diverse patient cohorts. They utilized mpMRI data, incorporating tumor delineations by radiologists and pathology reports. While internal cross-validation favored the deep-learning approach, the radiomics model demonstrated impressive performance with AUCs of 0.88, 0.91, and 0.65 on independent test sets, in contrast to the AUCs of 0.70, 0.73, and 0.44 for the deep-learning model.

Donisi et al. [22] conducted an investigation involving the integration of radiomics and machine learning techniques using a publicly available dataset to distinguish clinically significant from clinically non-significant prostate lesions. Their study demonstrated that tree-based algorithms achieved the highest evaluation metrics, consistently achieving accuracies exceeding 80%, with area-under-the-curve receiver-operating characteristics below 0.80. This underscores the utility of combining machine learning algorithms with radiomics in the context of routine, multiparametric magnetic resonance imaging for prostate cancer stratification.

Lastly, Zhang et al. [23] explored the value of radiomics signatures derived from biparametric magnetic resonance imaging (bp-MRI) in the preoperative prediction of prostate cancer grade, in comparison to visual assessments made by radiologists based on the Prostate Imaging Reporting and Data System Version 2.1 (PI-RADS V2). Their study revealed that radiomics signatures outperformed PI-RADS V2 scores in the preoperative prediction of prostate cancer grade. Furthermore, the concurrent utilization of radiomics signatures and PI-RADS V2 scores was shown to enhance diagnostic accuracy.

## Material and methods

In this research, we conducted a thorough analysis of T2W-transverse, T2W-sagittal, DWI, and ADC images. We employed various preprocessing techniques such as cropping, normalization, and enhancement to ensure the accuracy of the image data. Using the Pyradiomics software, we extracted handcrafted features from 52 images, which included fused images from eight different fusion techniques, as well as individual T2W-transverse, T2W-sagittal, DWI, and ADC images. To further enhance our analysis, we used an Autoencoder algorithm to extract deep features from the preprocessed images. We then applied a novel methodology called the "Tensor" paradigm to improve prediction performance. This framework allowed us to utilize various hybrid systems in conjunction with classifiers to predict the grading. Our overall goal was to investigate the potential benefits of using a combination of deep features and radiomics features based on a tensor approach, compared to the use of traditional hand-crafted radiomics features.

### Dataset and pre-processing

**Fig. 1:**
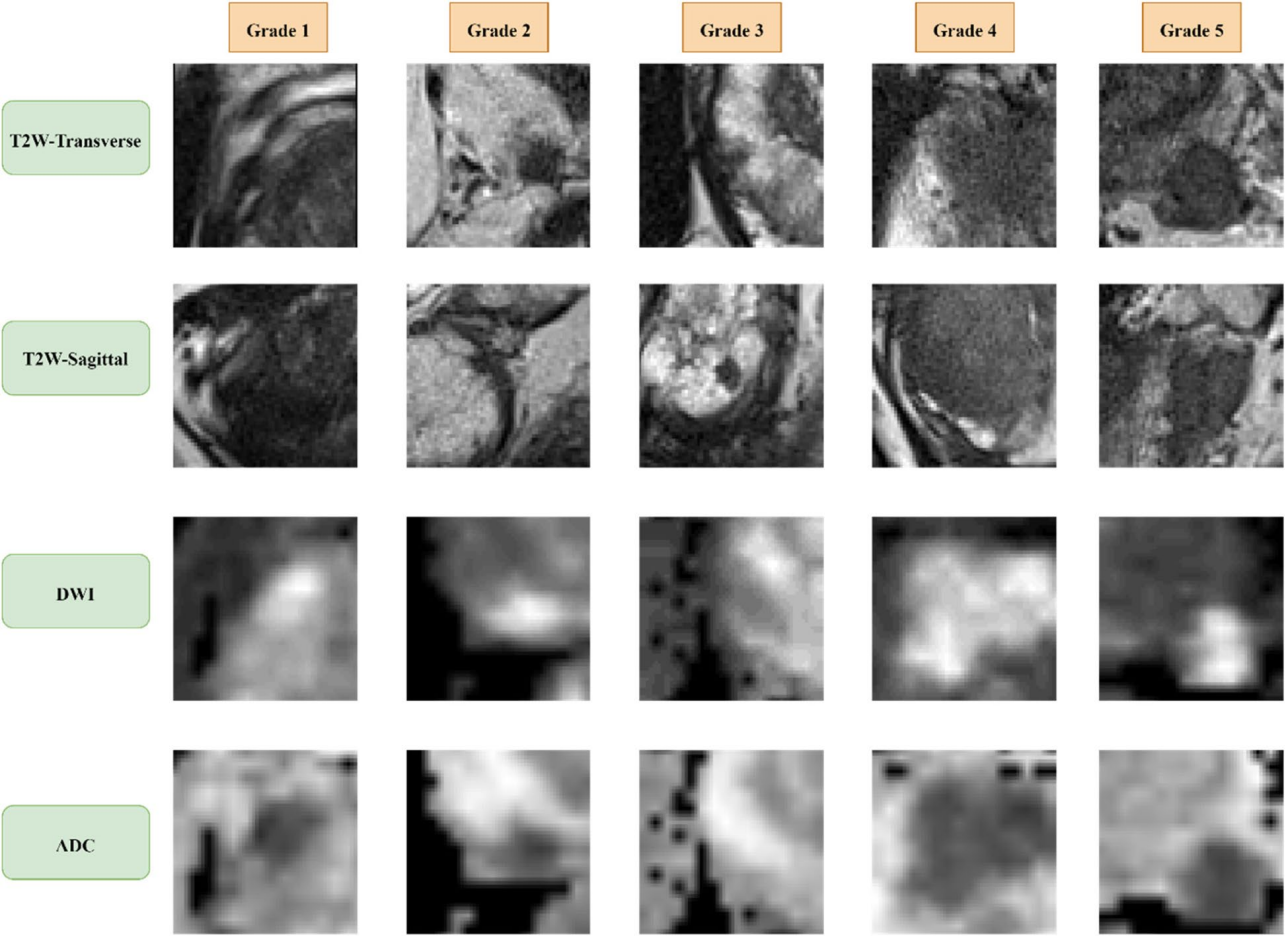
The 5 grade groups in T2W-transverse, T2W-sagittal, DWI, and ADC images

In this study, we utilized the PROSTATEx-2 dataset, which was previously used as the training dataset for the PROSTATEx-2 2017 challenge. An expert radiologist examined each MRI and assigned a PI-RADS score to any suspicious lesions, which were then biopsied and graded by a pathologist to serve as the standard for accuracy. We used the T2W, DWI, and ADC images from the dataset for the research, and the MR imaging parameters are summarized in Table 1. Figure 1 demonstrates the five grade groups in the T2W-transverse, T2W-sagittal, DWI, and ADC images. The dataset included 36 lesions in Grade Group 1, 40 in Grade Group 2, 20 in Grade Group 3, 8 in Grade Group 4, and 7 in Grade Group 5. To address the limitation of imbalanced data, we performed cross-validations on the PROSTATEx-2 training dataset and utilized the SMOTE technique, as explained in the data analysis section.

**Table 1:** MRI parameters

**Sequence**	**TR/TE** (ms)	**Slice Thickness** (mm)	**Matrix Size**	**Voxel Size** (mm)
T2W-transverse	5660/104	3	384 × 384	0.5 × 0.5 × 3
T2W-sagittal	5590/101	3.6	320 × 320	0.5625 × 0.5625 × 3.6
DWI	2700/63	3	128 × 84	2 × 2 × 3
ADC	2700/63	3	128 × 84	2 × 2 × 3

*TR/TE* Repetition Time/Echo Time, *FOV* Field of View

The ground truth of the PROSTATEx-2 dataset is a specific point in a (p, q, and r) coordinate voxel located approximately at the center of the tumor. To further analyze the data, we selected a region of interest (ROI) from both the DWI and ADC MRI volumes. This ROI was a 15 × 15 area that surrounded the ground truth point. Additionally, we selected an ROI of 60 × 60 from the T2W images, which also surrounded the ground truth point. The dimension of T2W images is different from the other modalities, as it is determined by the resolution of the image acquisition process. In the subsequent section, we used eight different fusion techniques to merge T2W, DWI, and ADC images, resulting in 48 images per patient and a total of 52 images for each patient. The use of multiple fusion techniques ensured that we were able to capture a wide range of information and gain a more detailed understanding of the subject being studied. Combining the T2W, DWI, and ADC images helped enhance the visibility of certain structures and improve diagnostic accuracy. Overall, the use of these fusion techniques greatly improved the quality and usefulness of the images obtained.

### Fusion of images

**Fig. 2:**
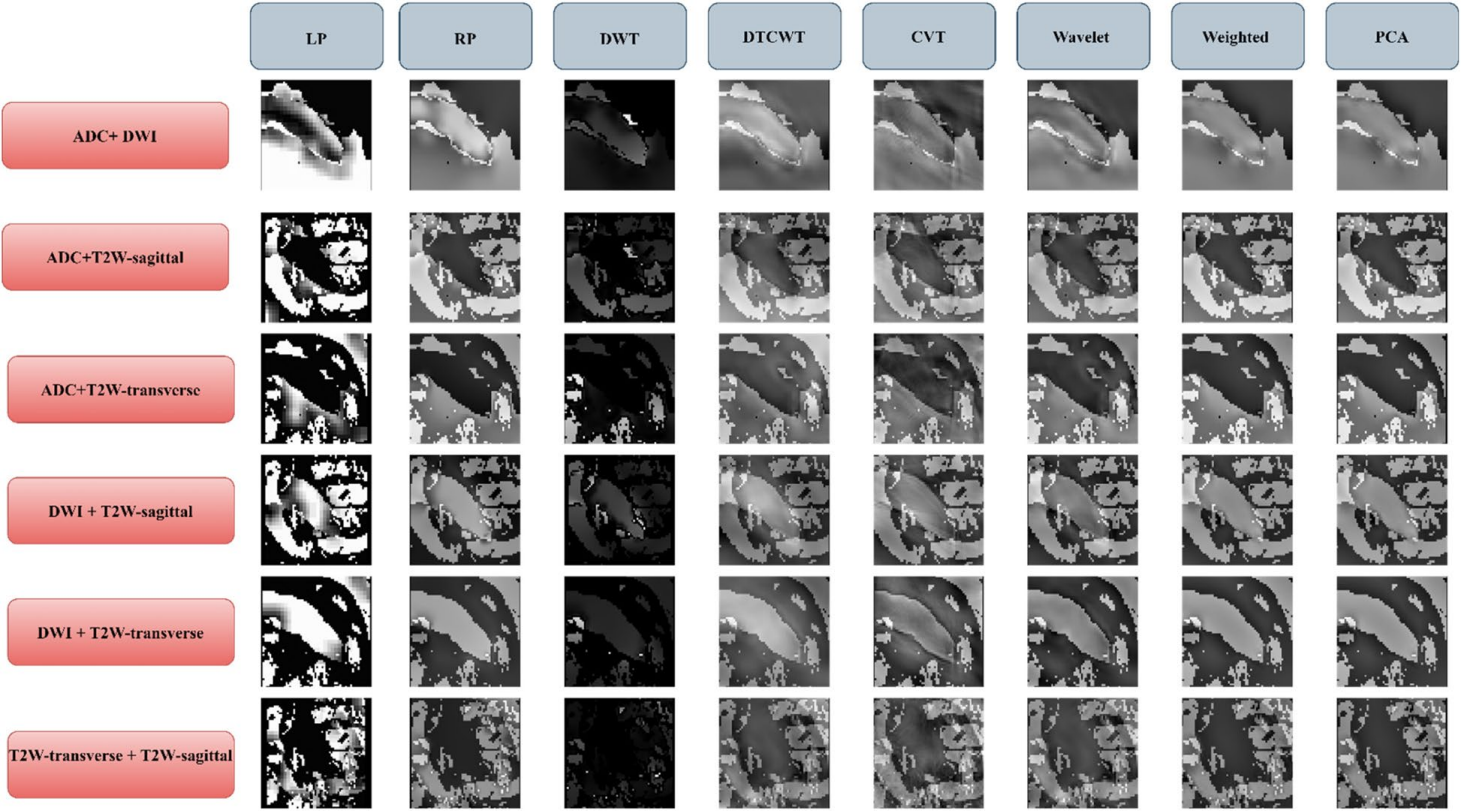
The fusion of T2W-transverse, T2W-sagittal, DWI, and ADC using 8 fusion techniques

In this research, we have employed various image-level fusion techniques to combine four different imaging modalities: T2W-transverse, T2W-sagittal, DWI, and ADC. These techniques include Laplacian Pyramid (LP), Ratio of the low-pass pyramid (RP), Discrete Wavelet Transform (DWT), Dual-Tree Complex Wavelet Transform (DTCWT), Curvelet Transform (CVT), Wavelet Fusion, Weighted Fusion, and Principal Component Analysis (PCA). Each technique has its own advantages and disadvantages, and the choice of method depends on the specific task and the characteristics of the images. LP is particularly useful in preserving fine details and edges. RP is useful for maintaining overall contrast and reducing noise. DWT is effective in preserving edges and fine details while reducing noise. DTCWT is suitable for complex image structures. CVT is effective in preserving fine details and edges while reducing noise. Wavelet Fusion is useful for preserving edges and fine details. Weighted Fusion is useful for maintaining overall contrast and reducing noise. PCA is effective in preserving the most important features of the image while reducing noise. Figure 2 illustrates the results of the fused images obtained from these techniques. The aim of this study is to improve the overall diagnostic accuracy and efficiency of medical imaging by utilizing these techniques.

### Radiomics feature extraction

In this study, we manually segmented prostate cancer images using the 3D Slicer software. The images were then analyzed, and radiomics features were extracted using the Pyradiomics library, an open-source tool in Python. We extracted a total of 107 quantitative radiomics features from each of the 52 patient images, including T2W-transverse, T2W-sagittal, DWI, ADC, and 48 fused images. These extracted features were classified into seven different categories: first-order features (18 features), shape features (14 features), gray-level dependence matrix (GLDM) features (14 features), gray-level co-occurrence matrix (GLCM) features (24 features), neighboring gray-tone difference matrix (NGTDM) features (5 features), gray-level size zone matrix (GLSZM) features (16 features), and gray-level run-length matrix (GLRLM) features (16 features).

### Deep feature extraction using Autoencoder

In this study, we propose a feature extractor based on an Autoencoder for deep feature learning in computer vision applications. Feature learning can be divided into two classes: supervised learning methods and unsupervised learning methods. Autoencoders, as a type of unsupervised neural network, have been proven to be effective in generating deep features from unlabeled data. The structure of an Autoencoder can be divided into two parts: the encoder and the decoder. The encoder compresses the original data into a lower-dimensional representation, while the decoder reconstructs the input from the compressed data. The input to the Autoencoder can be represented by x ∈ RN, where N is the dimension of the input data. The output of the encoder is a compressed representation of the input data that passes through the bottleneck of the network:

$${\mathrm{y}}_{\mathrm{encoded}}={\upsigma }_{1}({\mathrm{W}}_{1}\mathrm{x}+{\mathrm{b}}_{1})$$

Here, W_1_ represents the multiplication of the input layers to the bottleneck layer, and b1 is the corresponding bias term. σ_1_ denotes the activation function, which can be a sigmoid function or any other type of activation function. On the other hand, the output of the overall network can be obtained as follows:

$${\mathrm{y}}_{\mathrm{decoded}}={\upsigma }_{2}({\mathrm{W}}_{2}{\mathrm{y}}_{\mathrm{encoded}}+{\mathrm{b}}_{2})$$

where W_2_ represents the multiplication of the hidden layers from the bottleneck to the output, and b_2_ is the bias term. σ_2_ denotes the activation function, which can be a sigmoid function or any other type of activation function. The learning procedure starts by minimizing the following objective function J_N_:

$${\mathrm{J}}_{\mathrm{N}}=\frac{1}{2}\sum_{\mathrm{i}=1}^{\mathrm{N}}{\left({\mathrm{y}}_{\mathrm{decoded}}-\mathrm{x}\right)}^{2}$$

**Fig. 3:**
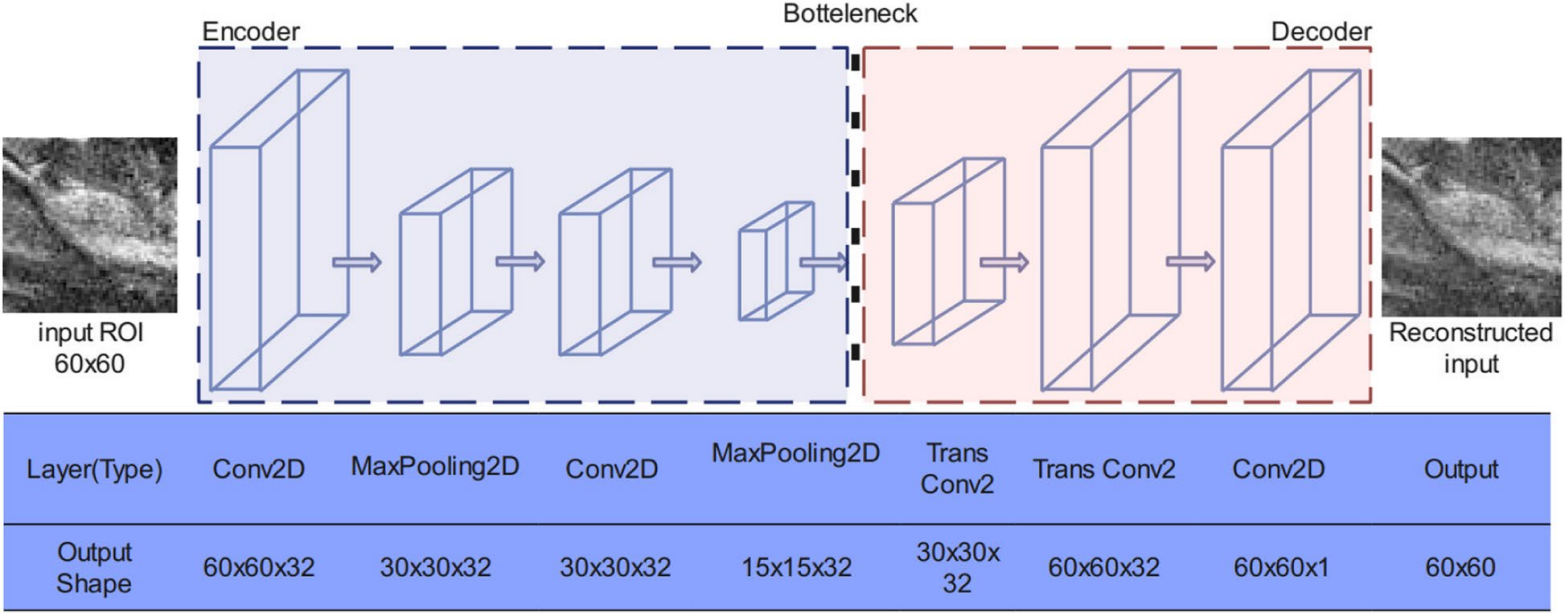
Structure of Autoencoder

The proposed feature extractor, as illustrated in Fig. 3, comprises four hidden layers in the encoder, including two 2D-convolutional layers, a 2D-MaxPooling layer, and another 2D-convolutional layer. The encoder’s output size is 32 × 15 × 15. The decoder consists of three hidden layers, which are two 2D-convolutional transpose layers and one 2D-convolutional layer. The decoder’s output size is 60 × 60. Rectified Linear Units (ReLU) were used as the activation function for all layers, except for the last layer of the decoder, where a sigmoid function was used instead. We utilized the backpropagation algorithm and k-fold cross-validation to minimize the objective function JN and converge to the best possible bounded value. Experimental results demonstrate the effectiveness of the proposed feature extractor in generating deep features.

### Feature sets and classification

**Fig. 4:**
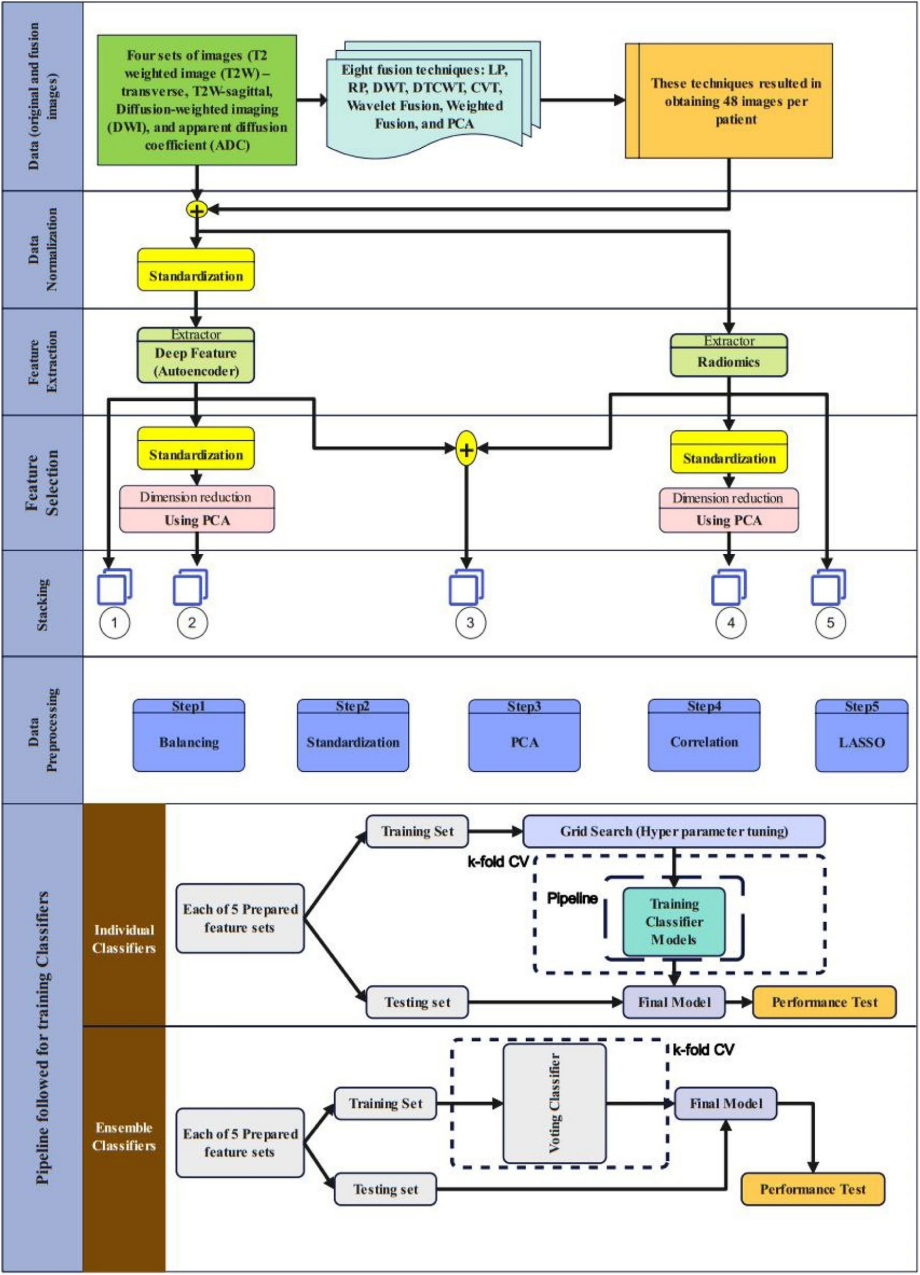
Flowchart of the proposed approach

The proposed approach’s structural design is illustrated in Fig. 4. The original dataset consisted of images from 111 patients, divided into four types: T2W-transverse, T2W-sagittal, DWI, and ADC. To expand the dataset, eight fusion methods (LP, RP, DWT, DTCWT, CVT, Wavelet Fusion, Weighted Fusion, and PCA) were used, resulting in 48 additional image sets. In the second step, the data was normalized and standardized to ensure equal contribution of each feature within a specific range [0, 1].

Figure 4 illustrates the use of five different feature sets to train the classifiers: all radiomics features, all deep features, radiomics features linked with PCA, deep features linked with PCA, and a combination of radiomics and deep features. The data was preprocessed in the sixth phase, which consisted of four steps: balancing, standardization, PCA (for feature sets 1, 3, and 5), correlation (using a heat map with a cutoff value of 0.8 to filter top correlated features), and Least Absolute Shrinkage and Selection Operator (LASSO) regression. The feature sets used were: 1) deep features only, 2) radiomics features only, 3) PCA on deep features (concatenated to form a file of size 111 × 260), 4) PCA on radiomics features (concatenated to form a file of size 111 × 107), and 5) a combination of deep features (converted from size (60, 60) to (111, 7200)) and radiomics features (concatenated to form a file of size 111 × 379964).

In the final classification phase, nine classifiers were used to classify different Gleason grades. Popular metrics, such as Logistic Regression, SVM, Gaussian Naive Bayes, KNN, Random Forest, Decision Tree + Bagging, and Gradient Boosting, were used to evaluate the classifiers’ performance. Additionally, two voting classifiers were employed: Voting 1 (consisting of Logistic Regression, SVM, Gaussian Naive Bayes, KNN, Random Forest, and Bagging) and Voting 2 (consisting of XGB Classifier, SVM, and Extra Tree Classifier). Grid search was used for hyperparameter tuning, and fivefold cross-validation was used for model validation for all classifiers except for the voting classifiers. Overall, the study presents a comprehensive approach for improving the classification of different Gleason grades in prostate cancer patients. The expansion of the original dataset and the use of various feature sets, fusion methods, and classifiers indicate the thoroughness of the study. The detailed explanation of the preprocessing steps and the evaluation metrics provide a clear understanding of the study’s methodology.

## Results

**Fig. 5:**
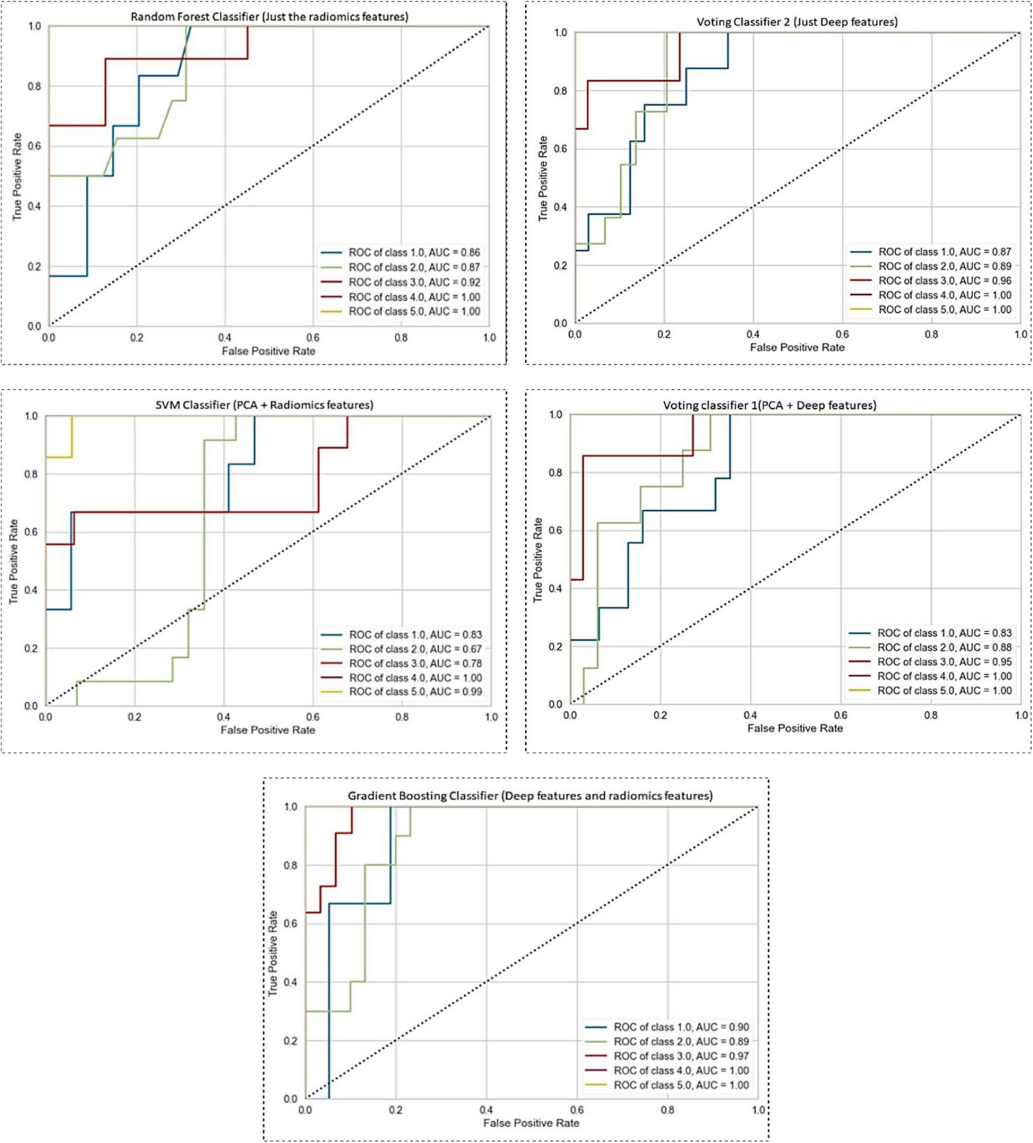
ROC curves for machine learning classifiers using different feature datasets

This section presents the results obtained from the proposed CAD approach. To assess the performance of the nine introduced classifiers, we employed balanced accuracy, Receiver Operating Characteristics (ROC) curve, and Area under ROC Curve (AUC) metrics. We conducted five sets of experiments for each classifier, using five corresponding prepared datasets, to evaluate the performance metrics. The results of our experiments are summarized in Table 2. The ROC curve provides a visualization of how well a classifier ranks positive samples over negative samples. The AUC can be interpreted as the probability that a positive sample receives a higher score than a negative sample. The corresponding best-achieved results of the ROC are depicted in Fig. 5.

**Table 2:** Classification performance metrics for the five datasets using nine classifiers

Classifier	Dataset Name	AUC	Balanced Accuracy
**SVM**	just the radiomics features	0.91 ± 0.013	0.76 ± 0.060
	just the deep features	0.94 ± 0.021	0.77 ± 0.065
	PCA + deep features	**0.94 ± 0.023**	**0.79 ± 0.048**
	PCA + radiomics features	0.92 ± 0.030	0.76 ± 0.060
	combination of deep features and radiomics features	0.94 ± 0.014	0.77 ± 0.045
**Logistic Regression**	just the radiomics features	0.89 ± 0.023	0.70 ± 0.057
	just the deep features	**0.93 ± 0.028**	**0.76 ± 0.064**
	PCA + deep features	0.88 ± 0.020	0.72 ± 0.052
	PCA + radiomics features	0.77 ± 0.029	0.61 ± 0.060
	combination of deep features and radiomics features	0.93 ± 0.016	0.77 ± 0.045
**Gaussian Naive Bayes**	just the radiomics features	0.91 ± 0.028	0.67 ± 0.074
	just the deep features	0.90 ± 0.027	0.67 ± 0.057
	PCA + deep features	0.88 ± 0.055	0.67 ± 0.107
	PCA + radiomics features	0.72 ± 0.046	0.39 ± 0.091
	combination of deep features and radiomics features	**0.92 ± 0.018**	**0.69 ± 0.046**
**KNN**	just the radiomics features	0.80 ± 0.043	0.66 ± 0.081
	just the deep features	0.77 ± 0.023	0.64 ± 0.037
	PCA + deep features	0.79 ± 0.025	0.65 ± 0.048
	PCA + radiomics features	**0.89 ± 0.040**	**0.70 ± 0.054**
	combination of deep features and radiomics features	0.79 ± 0.20	0.65 ± 0.029
**Random Forest**	just the radiomics features	0.93 ± 0.017	0.74 ± 0.043
	just the deep features	0.94 ± 0.028	0.78 ± 0.078
	PCA + deep features	0.92 ± 0.029	0.72 ± 0.081
	PCA + radiomics features	0.88 ± 0.028	0.68 ± 0.043
	combination of deep features and radiomics features	**0.94 ± 0.031**	**0.76 ± 0.086**
**Bagging + Decision Tree**	just the radiomics features	0.92 ± 0.016	0.70 ± 0.036
	just the deep features	0.93 ± 0.014	0.74 ± 0.048
	PCA + deep features	0.90 ± 0.036	0.69 ± 0.098
	PCA + radiomics features	0.84 ± 0.024	0.61 ± 0.058
	combination of deep features and radiomics features	**0.93 ± 0.029**	**0.73 ± 0.051**
**Gradient Boosting**	just the radiomics features	0.93 ± 0.019	0.73 ± 0.033
	just the deep features	0.94 ± 0.026	0.76 ± 0.082
	PCA + deep features	0.92 ± 0.030	0.74 ± 0.048
	PCA + radiomics features	0.88 ± 0.045	0.68 ± 0.076
	combination of deep features and radiomics features	**0.94 ± 0.021**	**0.78 ± 0.046**
**Ensemble classifier (Voting 1)**	just the radiomics features	0.92 ± 0.023	0.74 ± 0.033
	just the deep features	0.93 ± 0.021	0.78 ± 0.045
	PCA + deep features	0.92 ± 0.020	0.74 ± 0.067
	PCA + radiomics features	0.86 ± 0.031	0.60 ± 0.065
	combination of deep features and radiomics features	**0.94 ± 0.024**	**0.77 ± 0.055**
**Ensemble classifier (Voting 2)**	just the radiomics features	0.92 ± 0.023	0.74 ± 0.012
	just the deep features	**0.95 ± 0.020**	**0.78 ± 0.065**
	PCA + deep features	0.94 ± 0.024	0.76 ± 0.068
	PCA + radiomics features	0.90 ± 0.022	0.69 ± 0.033
	combination of deep features and radiomics features	0.94 ± 0.018	0.77 ± 0.033

The SVM classifier showed promising results when using deep features linked with PCA, with an AUC of 0.94 ± 0.023 and a balanced accuracy of 0.79 ± 0.048. The combination of deep features also performed well, with an AUC of 0.94 ± 0.014 and a balanced accuracy of 0.77 ± 0.045. Logistic regression performed best when using just the deep features, achieving an AUC of 0.93 ± 0.028 and a balanced accuracy of 0.76 ± 0.064. The combination of deep features also achieved high performance, with an AUC of 0.93 ± 0.016 and a balanced accuracy of 0.77 ± 0.045. Gaussian Naive Bayes had lower performance compared to other classifiers, with an AUC ranging from 0.72 ± 0.046 to 0.92 ± 0.018 and a balanced accuracy ranging from 0.39 ± 0.091 to 0.69 ± 0.046. KNN achieved high performance when using deep features linked with PCA, with an AUC of 0.89 ± 0.040 and a balanced accuracy of 0.70 ± 0.054. Random Forest showed high performance with the combination of deep features and radiomics features, achieving an AUC of 0.94 ± 0.031 and a balanced accuracy of 0.76 ± 0.086. When using just the deep features, Random Forest also achieved good results, with an AUC of 0.94 ± 0.028 and a balanced accuracy of 0.78 ± 0.07.

Ensemble classifiers, including Bagging with Decision Tree, Gradient Boosting, and Voting classifiers, all showed high performance when using a combination of deep features and radiomics features. The Voting classifiers showed higher performance when using just the deep features, with Voting 2 achieving the highest performance, with an AUC of 0.95 ± 0.020 and a balanced accuracy of 0.78 ± 0.065. Our results suggest that machine learning classifiers using imaging data can accurately classify data into different categories, with deep features showing higher performance than radiomics features alone.

Our findings indicate that the radiomics-only feature set did not yield better results in any of the classifiers mentioned above. This suggests that other features, such as clinical or demographic data, may be necessary to improve the performance of the classifiers. Table 3 presents a comparison of the results obtained in our study with those reported in other works.

**Table 3:** Comparison of AUC values of the proposed method with other ones developed using MR images

Study	Type of Image	Model	AUC (%)
**Castillo et al.** [18]	mpMRI	the deep-learning	88, 91 and 65
		the radiomics	0.70, 0.73 and 0.44
**Chaddad et al.** [24]	mpMRI	the radiomics	83.40, 72.71, and 77.35%
**Donisi et al.** [22]	MRI	the radiomics	80
**Gong et al**. [25]	MRI	the radiomics	79.4
**Wang et al**. [15]	ADC	the deep-learning	94
	T2W	the deep-learning	90
**Bertelli et al**. [26]	T2W	the deep-learning	0.75
**Our study**	mpMRI	the radiomics	92
		the deep-learning	95
		combination of deep features and radiomics features	94

## Discussion

Medical professionals are trained to visually diagnose malignant diseases using MRI scans. However, this method is subjective, error-prone, and slow, which limits its effectiveness as the burden on healthcare resources grows with the aging population [27–29]. Radiomic analysis, on the other hand, involves using machine learning algorithms to extract features from numerous images to automatically predict cancer grade with a precision and speed beyond the scope of human visual analysis [30, 31]. Previous studies have shown that radiomic features can assist in diagnosing cancer and offer insights into the heterogeneity of cancers[32]. Radiomics is also advantageous because it is automated, reducing human effort and cost, and preventing patient morbidity and mortality resulting from misdiagnosis or under/over-treatment [14, 24, 30]. Deep learning has the potential to improve the quality of Gleason grading by increasing consistency and providing expert-level grading independent of location [15]. Previous studies have applied deep learning to detecting cancer and Gleason grading of tissue microarrays, prostatectomies, and biopsies. However, these studies have been limited in scope, and a novel approach is needed to investigate the use of radiomics and deep features obtained from mpMRI to grade prostate cancer [33–35].

Our study proposes a new approach called multi-flavored feature extraction or tensor, which combines four mpMRI images using eight different fusion techniques to create 52 images or datasets for each patient. Our aim is to evaluate the effectiveness of this approach and compare it to traditional methods of grading prostate cancer. Our findings suggest that machine learning classifiers using imaging data can accurately classify data into different categories. Moreover, the use of deep features in conjunction with radiomics features shows higher performance than radiomics features alone. Among all classifiers evaluated, ensemble classifiers—particularly Voting 2—showed the highest performance. Finally, our results indicate that the radiomics-only feature set did not yield better results in any of the classifiers mentioned above.

Our study aimed to evaluate the performance of various machine learning classifiers in accurately classifying imaging data for the diagnosis of prostate cancer. We found that the SVM classifier and logistic regression achieved the best performance when using deep features alone or in combination with radiomics features, while Gaussian Naive Bayes had lower performance. Random Forest showed high performance using both types of features, and ensemble classifiers, particularly Voting 2, achieved the highest performance among all classifiers evaluated. These findings suggest that deep features may be more effective than radiomics features alone in accurately classifying prostate cancer. In contrast, Chaddad et al. [24] investigated whether radiomic features extracted from mpMRI scans can predict the Gleason score of prostate cancer patients. They found that certain radiomic features, including zone size percentage, large zone size emphasis, and zone size non-uniformity, were related to Gleason score groups and showed significant correlation. Using a Random Forest classifier, they were able to predict Gleason score groups with an average AUC ranging from 72.71% to 83.40%. Our study and Chaddad et al.’s work highlight the potential of using radiomic features extracted from imaging data as non-invasive biomarkers for the diagnosis and prediction of prostate cancer.

In the study conducted by Bulten et al. [36], the researchers aimed to investigate the potential of deep learning for automated Gleason grading of prostate biopsies. They developed a deep-learning system that could grade prostate biopsies following the Gleason grading standard. The system achieved high agreement with the reference standard and scored highly at clinical decision thresholds. In contrast, the results of our paper focus on the performance of different machine learning classifiers using imaging data to classify data into different categories. In particular, we utilize a novel approach called multi-flavored feature extraction, which involves combining radiomics and deep features in a tensor format. Furthermore, we explore the effectiveness of combining these tensor-based radiomics features with deep features to further improve the accuracy of the predictive model.

The study conducted by Gong et al. [25] aimed to explore the utility of radiomic features of the prostate gland in differentiating between Gleason scores (GS) of < 7, = 7, and > 7. They conducted a retrospective analysis of preoperative MRI data, clinical records, and postoperative pathological findings from a cohort of 489 patients with prostate cancer. Radiomic features were extracted in both 3D and 2D formats, obtained from manual segmentation of the 3D prostate gland and its maximum 2D layer on MRI, respectively. Sequence signatures were developed using multi-class linear regression (MLR), and 2D and 3D radiomic models were constructed by applying MLR to these sequence signatures. The 2D model demonstrated a C-index of 0.728 and an average area under the receiver operating characteristic curve of 0.794 in the validation dataset. In our study, we use a combination of handcrafted radiomic features and deep features extracted from preoperative MRI scans to predict the Gleason score. This approach captures different scales of features and considers the relationship between different features to improve the model’s performance. Both studies focus on predicting the Gleason score of prostate cancer patients using radiomic features extracted from MRI scans. However, our study employs a more advanced approach, which yields better results.

## Conclusion

In conclusion, our study has introduced a novel approach called multi-flavored feature extraction or tensor, which combines radiomics and deep features to predict the Gleason score of prostate cancer patients. Our results demonstrate that tensor deep features significantly outperform tensor radiomics features in predicting Gleason score. Furthermore, the use of Voting classifiers has shown higher performance when using just the deep features. Specifically, Voting 2 achieved the highest performance with an AUC of 0.95 ± 0.020 and a balanced accuracy of 0.78 ± 0.065. These findings suggest that quantitative imaging analysis, particularly the usage of tensor deep features and the combination of deep features and radiomics features, can be valuable in significantly enhancing Gleason score prediction performance. As such, this study may have important implications for improving the accuracy of prostate cancer diagnosis and ultimately contributing to better patient outcomes.

## Data Availability

Data and code about the results of this study will publicly share at: https://github.com/MASOUD-AJUMS/Exploring-the-Use-of-Radiomics-and-Deep-Features-for-Accurate-Grading-of-Prostate-Cancer-on-mpMRI-us.

## Abbreviations

DRE: Digital rectal examination
PSA: Prostate-specific antigen
TRUS: Transrectal ultrasound
GS: Gleason Score
GG: Grade Group
CAD: Computer-aided diagnosis
MRI: Magnetic resonance imaging
CNN: Convolutional Neural Network
mpMRI: Multiparametric MRI
T2W: T2 weighted image
DWI: Diffusion-weighted imaging
ADC: Apparent diffusion coefficient
ROI: Region of interest
LP: Laplacian Pyramid
RP: Ratio of the low-pass pyramid
DWT: Discrete Wavelet Transform
DTCWT: Dual-Tree Complex Wavelet Transform
CVT: Curvelet Transform
PCA: Principal Component Analysis
GLDM: Gray-level dependence matrix
GLCM: Gray-level co-occurrence matrix
NGTDM: Neighboring gray-tone difference matrix
GLSZM: Gray-level size zone matrix
GLRLM: Gray-level run-length matrix
ReLU: Rectified Linear Units
LASSO: Least Absolute Shrinkage and Selection Operator
ROC: Receiver Operating Characteristics
